# Supplementary material for: Antibacterial Activity of Solvothermal Obtained ZnO Nanoparticles with Different Morphology and Photocatalytic Activity against a Dye Mixture: Methylene Blue, Rhodamine B and Methyl Orange
Source: Int J Mol Sci. 2023 Mar 16;24(6):5677. doi: 10.3390/ijms24065677 (PMC10058279; doi:10.3390/ijms24065677)
Supplement: Supplementary file 1 [file ijms-24-05677-s001.zip › ijms-2161596-supplementary.pdf]

International Journal of *Molecular Sciences*

MDPI

Antibacterial activity of solvothermal obtained ZnO nanoparticles with different morphology and photocatalytic activity against a dye mixture: methylene blue, rhodamine B and methyl orange

Ludmila Motelica [1,2], Ovidiu–Cristian Oprea [1,2,3,4*], Bogdan-Stefan Vasile [1,2,3], Anton Ficai [1,2,3,4], Denisa Ficai [1,2,3], Ecaterina Andronescu [1,2,3,4], Alina Maria Holban [1,5]

1 National Research Center for Micro and Nanomaterials, University Politehnica of Bucharest, 060042 Bucharest, Romania
2 National Research Center for Food Safety, University Politehnica of Bucharest, Splaiul Independentei 313, 060042 Bucharest, Romania;
3 Faculty of Chemical Engineering and Biotechnologies, University Politehnica of Bucharest, 1-7 Polizu St., 011061 Bucharest, Romania;
4 Academy of Romanian Scientists, Ilfov Street 3, 050044 Bucharest, Romania
5 Microbiology & Immunology Department, Faculty of Biology, University of Bucharest, 077206 Bucharest, Romania
* Correspondence: ovidiu.oprea@upb.ro or ovidiu73@yahoo.com;

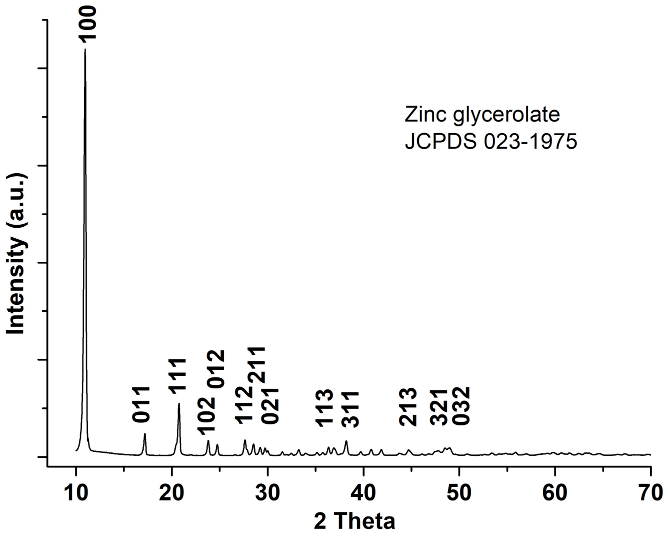

Figure S1. XRD of obtained zinc glycerolate (JCPDS 023-1975)

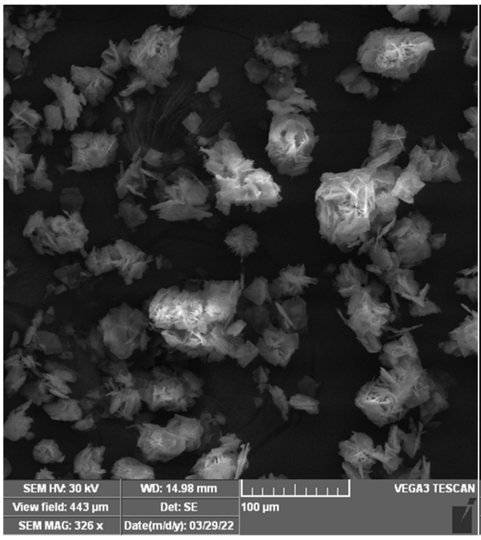

(**a**)

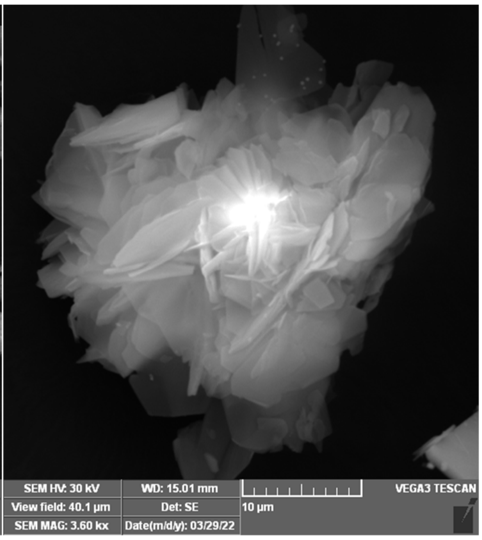

(**b**)

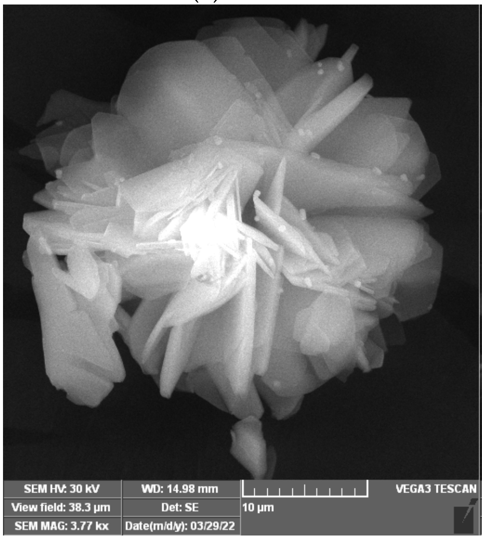

(**c**)

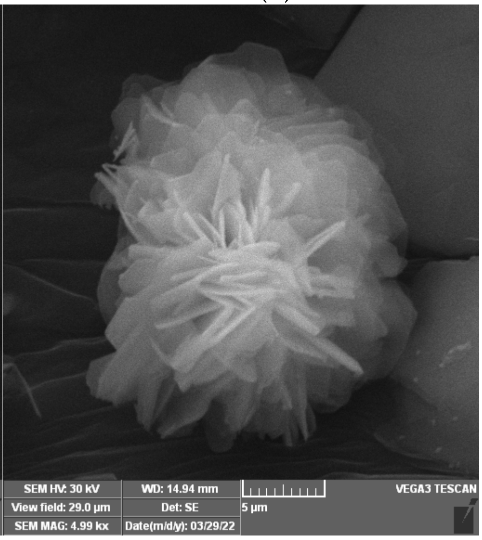

(**d**)

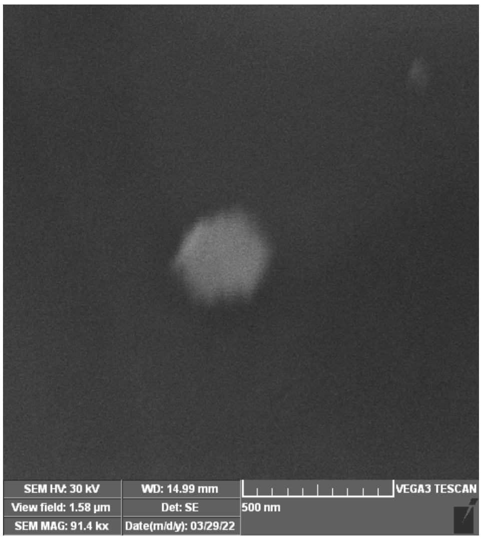

(**e**)

Figure S2. SEM images of zinc glycerloate rose-like structure (**a-d**); 200 nm hexagonal particles (**e**)

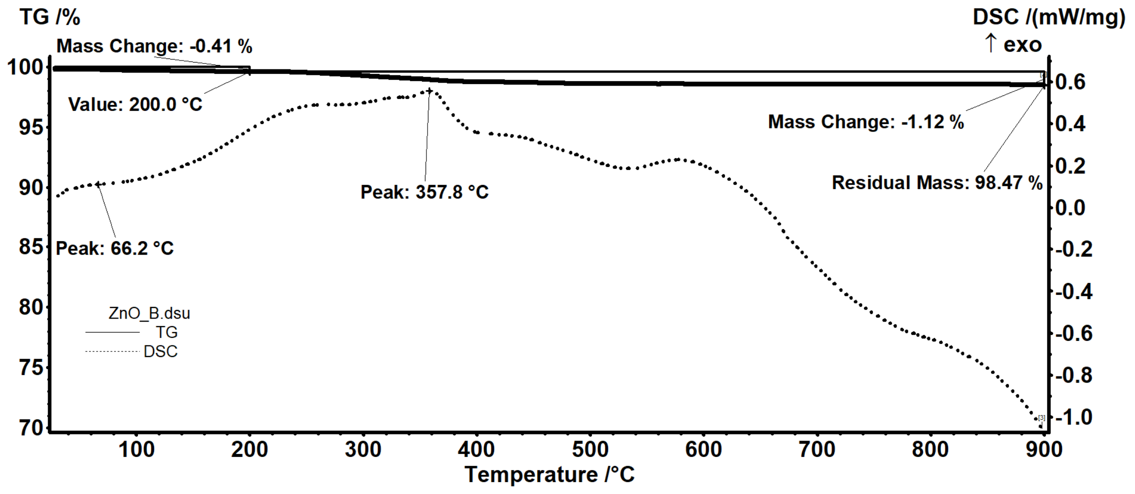

(a)

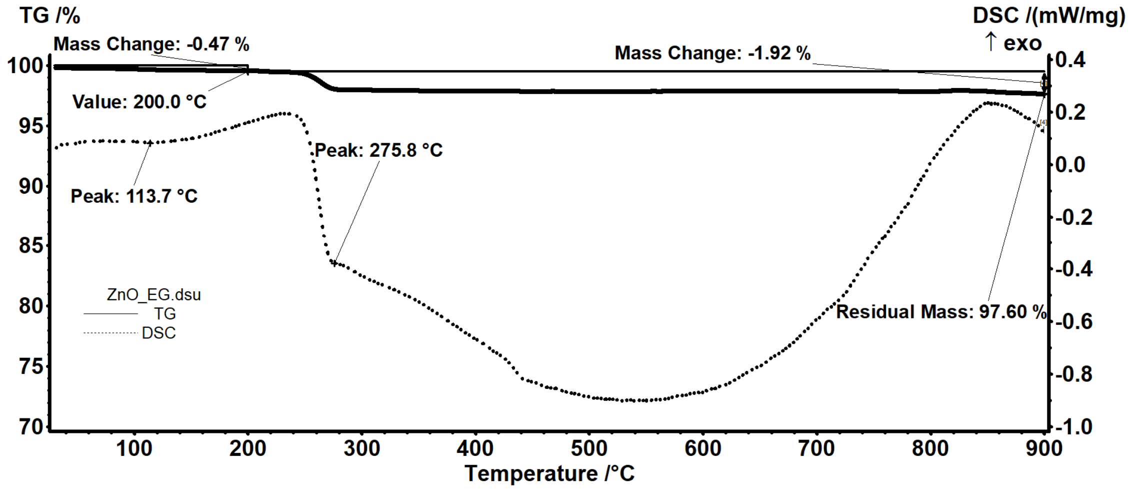

(b)

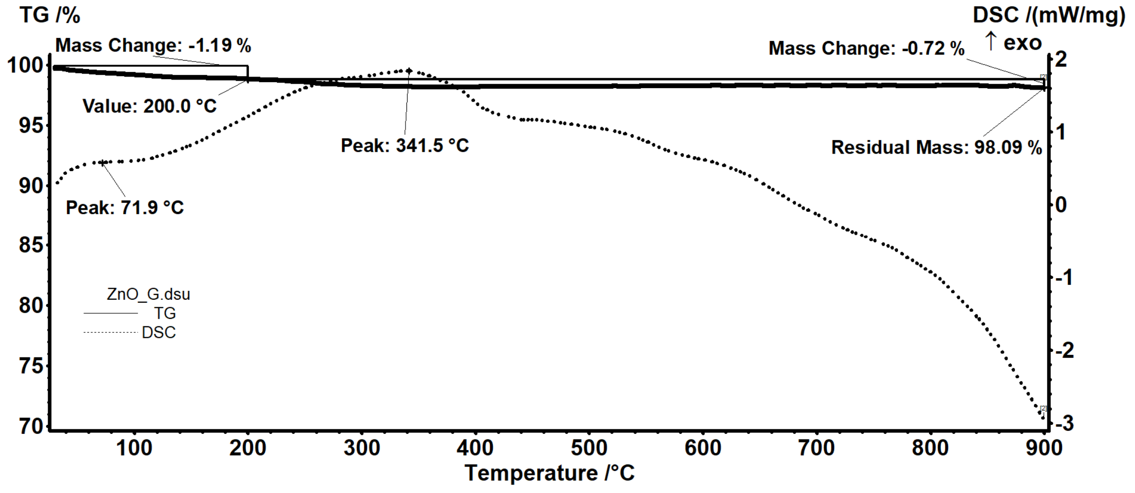

(c)

Figure S3. TG-DSC analysis for ZnO_B sample obtained in n-butanol (**a**); ZnO_EG sample obtained in ethylene glycol (**b**); ZnO_G sample obtained from zinc glycerolate in water (**c**)

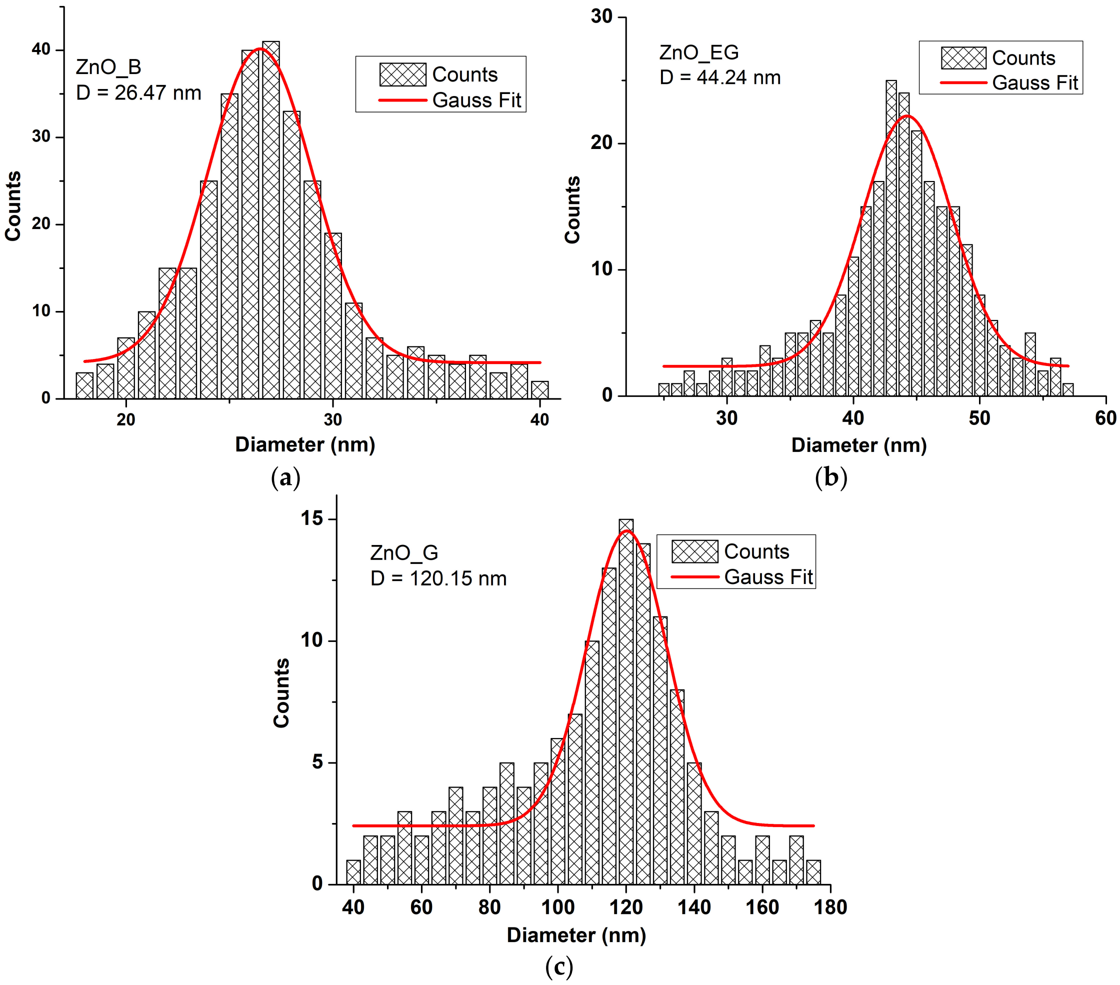

Figure S4. Size distribution for ZnO nanoparticles: (**a**) ZnO_B obtained in n-butanol; (**b**) ZnO_EG obtained in ethylene glycol and (**c**) ZnO_G obtained from zinc glycerolate in water
